# Pharmacogenomics of response to statin treatment and susceptibility to statin-induced adverse drug reactions in Asians: a scoping review

**DOI:** 10.2478/abm-2023-0050

**Published:** 2023-10-09

**Authors:** Hui-Yin Yow, Sharina Hamzah, Nusaibah Abdul Rahim, Vijayaprakash Suppiah

**Affiliations:** 1 Department of Pharmaceutical Life Sciences, Faculty of Pharmacy, Universiti Malaya, Kuala Lumpur 50603, Malaysia; 2 School of Pharmacy, Faculty of Health and Medical Sciences, Taylor's University, Selangor 47500, Malaysia; 3 Medical Advancement for Better Quality of Life Impact Lab, Taylor's University, Selangor 47500, Malaysia; 4 Department of Clinical Pharmacy and Pharmacy Practice, Faculty of Pharmacy, Universiti Malaya, Kuala Lumpur 50603, Malaysia; 5 Clinical and Health Sciences, University of South Australia, Adelaide, SA 5001, Australia; 6 Australian Centre for Precision Health, University of South Australia, Adelaide, SA 5001, Australia

**Keywords:** cholesterol, HMG-CoA reductase inhibitors, HMG-CoA statins, myotoxicity, pharmacogenomics

## Abstract

**Background:**

Statins are the most widely used lipid-lowering agents for patients with hyperlipidemia. However, interindividual variations in efficacy and risk of adverse drug reactions to statin treatment have been widely reported. Ethnicity is well known to be one of the contributing factors to this variation, particularly among Asians.

**Objectives:**

To identify genetic variants associated with statin treatment responses among Asian populations with a focus on four commonly prescribed statins: atorvastatin, rosuvastatin, simvastatin, and pravastatin.

**Methods:**

A literature search was conducted in Medline and Embase databases. Studies published from 2008 to 2021 were included. The title and abstract of each article were screened by two reviewers and verified by another two reviewers. Data charted include information on authors, year of study, study population, statin studied, gene studied, study findings, and data of significant statistical value.

**Results:**

A total of 35 articles were included from the 1,939 original studies related to treatment efficacy and 5 articles out of the 284 original studies related to adverse effects. Genetic variants in transmembrane transporters, cytochrome P450 isoenzymes, and apolipoproteins are the most extensively studied among Asian populations, with a main focus on ethnic Chinese. However, Asia consists of genetically different populations, and the results of this review indicated that there is a paucity of studies on other ethnic groups within Asia.

**Conclusions:**

Considering the ethnicity of patients could provide a potential value to personalized medicine in statin therapy.

Statins are the most widely used lipid-lowering drugs across the globe for the treatment of hyperlipidemia. The cholesterol-lowering activity of statins is attributed to the inhibition of 3-hydroxy-3-methyl-glutaryl-coenzyme A (HMG-CoA) reductase enzyme. This inhibition of enzyme activity increases the level of low-density lipoprotein (LDL) receptors, thereby increasing the uptake and degradation of LDL-cholesterol (LDL-C), reducing the synthesis and accumulation of cholesterol and decreasing the secretion of lipoprotein [1]. Statin therapy-induced reduction in blood concentrations of LDL-C has been shown to decrease the rates of coronary heart disease in different populations [2, 3]. This evidence is robust for both primary and secondary prevention, in men and women, in older and younger people, in people with and without diabetes mellitus, in people with and without hypertension, and in people with higher or lower levels of baseline LDL-C, with the possible exception of those with end-stage renal disease, at risk of having an atherosclerotic cardiovascular disease event [4].

Among the statins, rosuvastatin is the most potent and delivers the most significant reduction in LDL-C, with 5 mg of rosuvastatin being equivalent to up to 10 mg of atorvastatin, up to 40 mg of simvastatin, and up to 80 mg of pravastatin, respectively [5]. Apart from the potency of different statins, the ethnicity of patients, particularly Asian ancestry, has been identified as an important factor in determining statin doses [6]. Scoping review is used to chart the literature available and to identify potential research gaps. Thus, the aims of this scoping review were: (i) to identify the genetic variants associated with statin treatment responses among Asian populations, and (ii) to explore the effects of these genetic variants on drug efficacy and susceptibility to statin-induced adverse drug reactions (ADR). This review focused on pharmacogenomic studies on the four commonly prescribed statins: atorvastatin, rosuvastatin, simvastatin, and pravastatin and highlighted the research gaps that should be considered in future pharmacogenomic research.

## Methods

### Search strategy

The literature search was conducted in two databases, Medline and Embase, using free-text terms. The Boolean operator “OR” was used to group search terms into three main subjects. The first subject included terms relating to pharmacogenetics – “pharmacogen*”, or “genetic*”, or “genomic*”, or “genotype”; secondly, terms related to statins – “statins”, or “atorvastatin”, or “rosuvastatin” or “simvastatin” or “pravastatin”; and lastly terms relating to the treatment responses – “treatment response” or “efficacy” or “response”. In terms of adverse effects, the first two subjects used were similar, the subject used included the terms “adverse effect” or “adverse drug reaction”. Each of the groups of search terms was searched simultaneously with the “AND” Boolean operator to identify original research articles published up to December 2021 on pharmacogenomics of statin treatment responses, which focused on atorvastatin, rosuvastatin, simvastatin, and pravastatin. The search was limited to articles published in the English language with human subjects. Abstracts or conference proceedings were included if the content was not published before.

### Screening and study selection

**Figure 1.**
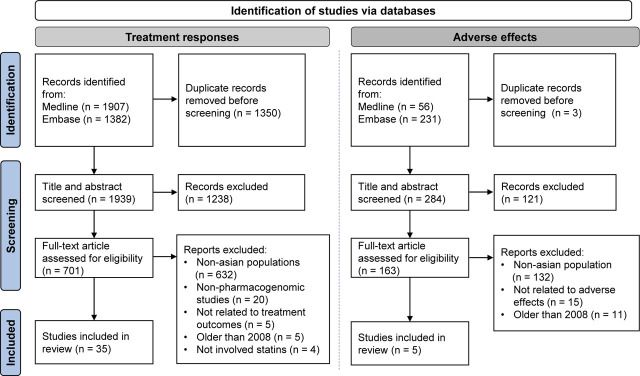
Flow diagram of selection process for scoping review of pharmacogenomics of statin treatment outcomes in Asians based on Preferred Reporting Items for Systematic Reviews and Meta-Analyses (PRISMA).

A total of 1907 papers from Medline and 1,382 papers from Embase were identified for treatment response to statins. After removing duplicates, 1939 original papers remained (**Figure 1**). For adverse effects, 56 papers from Medline and 231 papers from Embase were identified equating to 284 individual articles after removing duplicates. Compilation and elimination of duplicates was conducted using the referencing software “Mendeley”. The title and abstract of each article were reviewed by researchers YHY and VS and verified by SH and NAR. Only papers from 2008 to December 2021 and studies carried out in Asian populations were included. Reference lists of past reviews were checked for other papers of interest to ensure that the search was all inclusive.

Articles that did not answer the research question were excluded. Articles were excluded if they contained: pharmacogenomic studies of statins other than atorvastatin, rosuvastatin, simvastatin, and pravastatin; pharmacogenomic studies with subjects of unreported ethnicities; non-pharmacogenomic studies (such as pharmacokinetic studies, gene expression studies, or cell culture studies); and population genotyping studies not involving statins. The study protocol was prospectively registered with Open Science Framework (OSF) registries (https://osf.io/sqp6x).

### Data extraction

Due to the nature of the scoping review, the quality of the individual studies was not assessed in the present study. Extracted data included information on authors, publication year, study population, statin(s) studied, gene(s) studied, study findings, and data of significant statistical value. Four reviewers (YHY, SH, NAR, and VS) independently extracted data into the data extraction form. Any conflicts were resolved within the team.

### Summarizing and reporting results

The data on (i) genetic variants studied in statin treatment response and (ii) genetic variants studied in statin-induced ADRs were summarized. The Preferred Reporting Items for Systematic reviews and Meta-Analyses extension for Scoping Reviews (PRISMA-ScR) was used as a checklist in documenting the rationale, methodology, and findings of the scoping review [7].

## Results

A total of 40 original manuscripts were included in this review: 35 articles for treatment response and 5 articles for statin-induced adverse effects.

### Genetic variation in statin treatment response

**Figure 2.**
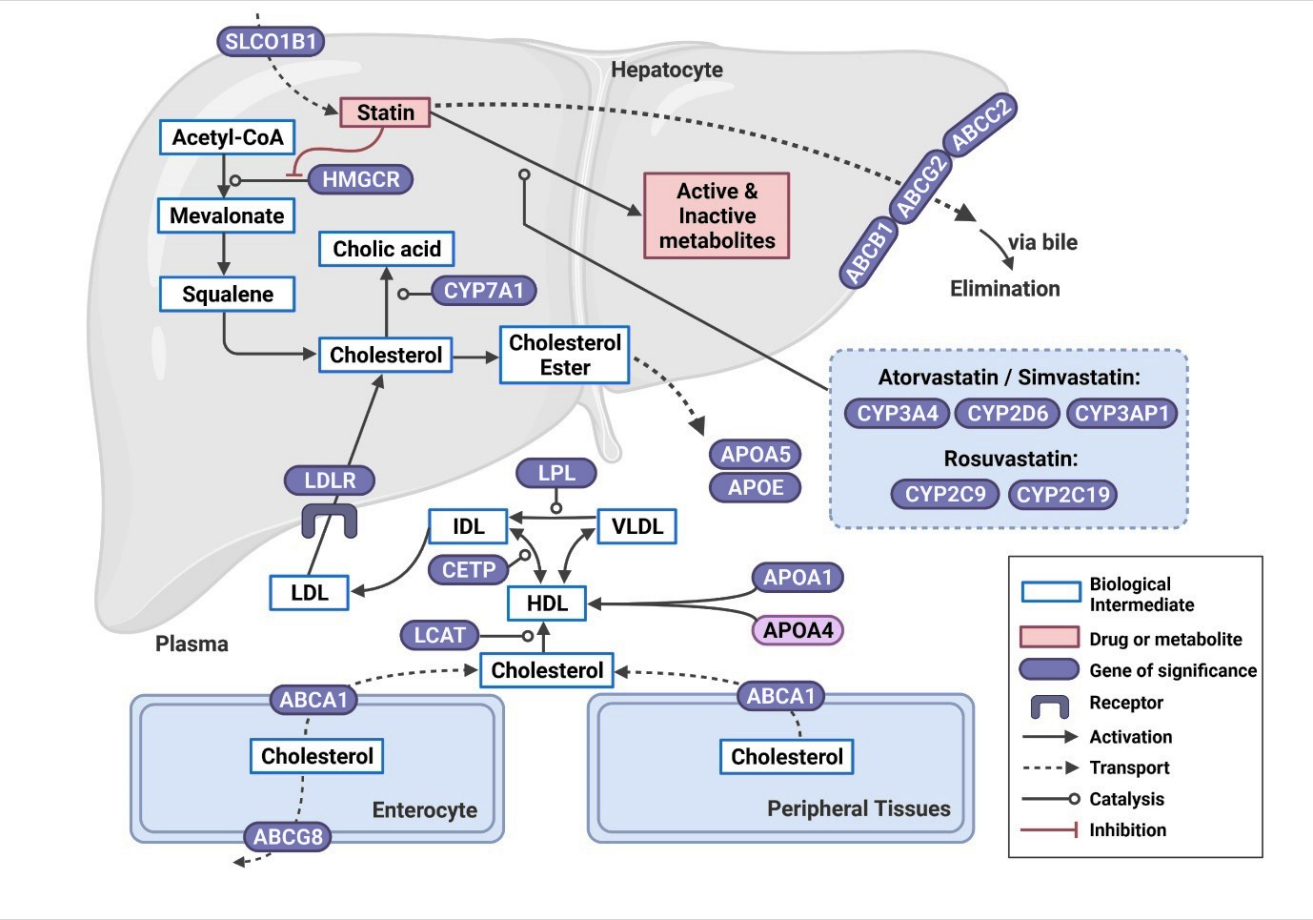
Gene polymorphisms significantly affecting the therapeutic response of statins (atorvastatin, rosuvastatin, simvastatin, and pravastatin) in Asian populations. Statins are transported into hepatocytes via active transport through SLC transporters and metabolized mainly by CYP enzymes and eliminated by efflux pumps, ABC proteins. In terms of mechanism of action, statins inhibit HMGCR, which is the step-limiting step in cholesterol synthesis. Created with BioRender.com. Adapted from PharmGKB [8]. ABC, ATP-binding cassette; APO, apolipoprotein; APOA5, Apolipoprotein A-V; APOE, apolipoprotein E; CETP, cholesterol ester transfer protein; CYP, cytochrome P450; CYP7A1, cholesterol 7-α-hydroxylase; HDL, high-density lipoprotein; HMGCR, HMG-CoA reductase; IDL, intermediate-density lipoprotein; LCAT, lecithin-cholesterol acyltransferase; LDL, low-density lipoprotein; LDLR, LDL receptor; LPL, lipoprotein lipase; SLC, solute carrier; VLDL, very-low-density lipoprotein.

Among the 35 papers investigating treatment response, 28 papers were studies investigating a single statin (rosuvastatin, n = 9; simvastatin, n = 10; atorvastatin, n = 8; pravastatin, n = 1), 6 papers investigated multiple statins, and 1 paper did not specify the statin(s). The parameters used to determine the treatment response in the included studies were the changes in total cholesterol (TC), triglycerides (TG), LDL-C, and high-density lipoprotein cholesterol (HDL-C) levels. The data extracted from these papers are summarized in **Table 1**. **Figure 2** depicts the important genes that have been significantly associated with response to statin treatment [8].

**Table 1.** Genetic polymorphisms affecting pharmacokinetics of statins among Asians

**Gene**	**Variant**	Statin^*^	**Ethnic**	**Allele/Genotype/Haplotype frequency (%)**	**Study population**	**Effect**	**Ref.**
**Transporter**							

SLCO1B1	A388G rs2306283	ATV	Indian	A: 43 G: 57	177 DL	Greater LDL reduction (AA)	[14]
		SIM	Thai	AA: 7.7 AG: 34.8 GG: 57.5	391 DL	NS	[10]
		RSV	Chinese	AA: 6.3 AG: 35.8 GG: 57.9	291 DL	NS	[11]
		RSV, SIM	Chinese Han	AA: 4.8 AG: 35.1 GG: 60.1	247 CAD, HCVD	NS	[12]
	T521C rs4149056	SIM	Thai	TT: 78.5 TC: 19.4 CC: 2.1	391 DL	NS	[10]
		RSV	Chinese	TT: 75.4 TC: 22.8 CC: 1.8	291 DL	NS	[11]
		RSV, SIM	Chinese Han	TT: 74.7 TC: 23.3 CC: 2.0	247 CAD, HCVD	NS	[12]
	G> A rs4149081	RSV, SIM	Chinese Han	GG: 22.7 GA: 53.4 AA: 23.9	247 CAD, HCVD	Greater LDL reduction (A allele)	[12]
	C463A rs11045819	ATV	Indian	C: 97 A: 3	177 DL	NS	[14]
	T89595C rs4363657	SIM	Thai	TT: 41.2 TC: 43.2 CC: 15.6	391 DL	NS	[10]
SLC10A1	^*^2 rs2296651	RSV	Chinese	^*^1^*^1: 84.0 ^*^1^*^2: 14.9 ^*^2^*^2: 1.1	291 DL	NS	[11]
ABCA1	R219K	PRV	Chinese	RK: 46.6 RR: 38.3 KK: 15.1	365 CAD	Greater HDL increment (KK)	[26]
ABCC2	−24 C>T rs717620	SIM	Chinese Han	GG: 60.7 GA: 31.4 AA: 7.9	318 DL	Low response of HDL elevation (A allele)	[17]
		ATV	Chinese Han	NA	318 HCVD	Significant different in percentage of LDL change	[18]^#^
ABCB1	G2677T rs2032582	ATV	Indian	G: 35 T: 65	177 DL	Greater LDL reduction (T allele)	[14]
	C3435T rs1045642	ATV	Indian	C: 39 T: 61	177 DL	Greater LDL reduction (T allele)	[14]
	rs2235013, rs2235033, rs1128503, rs10276036	ATV	Chinese Han	NA	318 HCVD	Significant different in percentage of LDL change	[18]^#^
ABCG2	C421A rs2231142	RSV	Chinese	CC: 46.8 CA: 39.1 AA: 14.1	291 DL	Lower LDL reduction (CC)	[11]
		RSV	Chinese	CC: 50 CA: 35.7 AA: 14.3	386 HCVD, FH, RA	Greater LDL reduction (AA/CA)	[20]
		RSV	Chinese Han	CC: 51.8 CA: 35.4 AA: 12.8	305 DL	Greater LDL reduction (AA)	[21]
	G34A rs2231137	RSV	Chinese	GG: 46.7 GA: 43.2 AA: 10.1	386 HCVD, FH, RA	Greater LDL reduction (GG)	[20]
ABCG5	rs6720173	ATV	Indian	C: 75 G: 25	177 DL	NS	[14]
ABCG8	rs4148222	ATV	Chinese Han	CC: 44.9 CT: 43 TT: 12.1	107 DL	Lower baseline HDL (CC)	[22]
	D18H rs11887534	ATV	Indian	DD: 88.7 DH, HH: 11.3	213 CAD; 220 H	NS	[25]
	C1199A	ATV	Chinese Han	CC: 75.1 CA, AA: 24.9	181 DL	NS	[23]
	rs11887534, rs4148217, rs4148214, rs17606027, rs4952689, rs4953028	ATV	Chinese Han	-	107 DL	NS	[22]
	Haplotypes	V	Chinese	GCGACTGCC: 34.0 GCGATCGCC: 28.3 GCGATTGCC: 8.9 ATTATCGAC: 5.3	386 DL	Greater LDL reduction (ATTATCGAC haplotype)	[24]

**Cytochrome P450 and related enzymes**							

CYP2C9	rs1934967	V	Chinese	CC: 66.2 CT: 30.1 TT: 3.7	386 DL	NS	[24]
	^*^1	RSV	Chinese	^*^1^*^1: 90.4 ^*^1^*^3, ^*^3^*^3: 9.6	218 DL	Greater TC and LDL reduction (^*^1^*^3/^*^3^*^3)	[33]
	^*^3 rs1057910	RSV	Chinese	^*^1^*^1: 93.8 ^*^1^*^3: 5.8 ^*^3^*^3: 0.4	291 DL	NS	[11]
CYP2C19	^*^2 (rs4244285;	ATV	Chinese Han	NA	192 IS	NS	[32]
	^*^3 (rs4986893)	RSV	Chinese	^*^1^*^1: 37.2 ^*^1^*^2, ^*^1^*^3: 54.4 ^*^2^*^2, ^*^2^*^3: 8.4	291 DL	NS	[11]
	rs10786172	V	Chinese	AA: 65.9 AG: 30.2 GG: 3.9	386 DL	NS	[24]
	^*^1	RSV	Chinese	^*^1^*^1, ^*^1^*^2, ^*^1^*^3: 51 ^*^2^*^2, ^*^2^*^3, ^*^3^*^3: 49	49 H	Greater TG reduction (poor metabolisers: ^*^2^*^2/^*^2^*^3/^*^3^*^3)	[34]
CYP2D6	^*^10(C188T)	SIM	Chinese (Ningxia Hui)	CC: 33 CT: 39 TT: 28	200 DL	Greater TC and LDL reduction (CC)	[35]
	^*^10 rs1065852	ATV	Chinese Han	GG: 16.7 GA: 49.5 AA: 33.8	192 IS	Greater LDL reduction (G allele)	[32]
CYP3A4	rs2242480	ATV	Chinese Han	CC: 51.0 CT: 44.3 TT: 4.7	192 IS	Greater LDL reduction (C allele)	[32]
	^*^1G	SIM	Chinese	^*^1^*^1: 53.4 ^*^1^*^1G: 39.9 ^*^1G^*^1G: 6.7	273 DL	NS	[30]
		SIM, ATV	Chinese	^*^1^*^1: 49.31–52.3 ^*^1^*^1G: 40.70–45.6 ^*^1G^*^1G: 5.07–7.1	423 DL	Greater TC reduction (^*^1G^*^1G) with ATV; NS with SIM	[31]
	^*^22 rs35599367	SIM	Chinese	No variant allele	273 DL	NA	[30]
	rs2740574	ATV	Indian	A: 97 G: 3	177 DL	Greater LDL reduction (AA)	[14]
	rs4986910	ATV	Indian	T: 99 C: 1	177 DL	NS	[14]
CYP3A5	^*^3 6986G>A rs776746	SIM	Chinese	^*^1^*^1: 6.7 ^*^1^*^3: 39.8 ^*^3^*^3: 53.5	273 DL	NS	[30]
		ATV	Chinese	^*^1^*^1: 4.5 ^*^1^*^3: 43.5 ^*^3^*^3: 52.0	179 DL	NS	[36]
		ATV	Indian	A: 26 G: 74	177 DL	NS	[14]
	rs4646450, rs3800959, rs776746	ATV	Chinese Han	-	107 DL	NS	[22]
CYP3AP1	^*^3 -44G>A rs217718	SIM	Chinese Han	^*^3^*^3: 44.4 ^*^1^*^3: 46.0 ^*^1^*^1: 9.6	202 DL	Greater LDL reduction (^*^3^*^3 women)	[37]
		ATV	Chinese Han	^*^3^*^3: 54.8 ^*^1^*^3: 41.8 ^*^1^*^1: 3.4	177 DL	Greater TC reduction (^*^3^*^3 women)	[37]
CYP4F2	rs2108622	ATV	Chinese Han	NA	192 IS	NS	[32]
PPARA	A>G rs4823613	SIM	Chinese	AA: 57.4 AG: 37.4 GG: 5.2	273 DL	NS	[30]
POR	^*^28	ATV	Chinese	CC: 31.8 CT: 53.1 TT: 15.1	179 DL	Lower mean LDL (TT)	[36]

**Apolipoproteins**							

APOA5	T-1131C rs662799	V	Chinese	TT: 46.7 TC: 46.7 CC: 6.6	195 DL, HCVD	Greater LDL reduction (TT); Greater HDL and TG reduction (TT; only ATV & SIM)	[44]
		RSV	Chinese	TT: 47.8 TC: 41.3 CC: 10.9	386 DL, FH	NS	[45]
APOE	E2/E3/E4 rs7412, rs429358	RSV	Chinese	E2E2, E2E3: 8.7 E3E3: 69.0 E4E4, E4E3: 22.3	386 DL, FH	NS	[45]
		SIM	Thai	E2E2, E2E4: 0 E2E3: 14.22 E3E3: 62.22 E3E4: 20.44 E4E4: 3.11	225 DL	Greater TC and LDL reduction (APOE2, APOE3 carriers)	[47]
	rs4420638	RSV	Chinese	AA: 77.2 AG: 20.7 GG: 2.1	386 HCVD, FH, RA	Greater LDL reduction (AA/AG)	[20]
APOA1	G75A	PRV	Chinese	GG: 45.8 AA: 14.5 GA: 39.7	97 DL	Greater TC and LDL reduction (AA/GA)	[48]

**3-hydroxy-3-methyl-glutaryl-coenzyme A reductase**							

HMGCR	rs3931914	V	Chinese	GG: 47.4 GC: 42.5 CC: 10.1	386 DL	NS	[24]
	rs12916	V	Chinese	CC: 30.1 CT: 50.9 TT: 19.0	386 DL	Greater LDL reduction (CT/TT)	[24]
	Haplotypes	V	Chinese	GCGTTCA: 44.2 CCGTCCA: 23.5 GTGACTA: 19.1 CCATCCA: 7.4	386 DL	Higher LDL (CCGTCCA haplotype)	[24]
	rs3846662	ATV	Korean	GG: 54.2 AA: 45.8	24 H	Higher LDL (GG)	[50]

**LDL receptor and related genes**							

LDLR	rs1433099	RSV	Chinese	CC: 52.5 CT: 40.0 TT: 7.5	386 HCVD, FH, RA	Greater LDL reduction (CC/CT)	[20]
PCSK9	I474V rs562556	SIM	Thai	II: 96.4 IV: 3.6 VV: 0	225 DL	Greater LDL reduction (IV)	[47]
	R46L rs11591147; E670G rs505151	SIM	Thai	NA	225 DL	NA	[47]
SREBF1	rs9902941	V	Chinese	CC: 84.4 CT: 15.1 TT: 0.5	386 DL	Greater LDL reduction (CT/TT)	[24]

**Enzymes involved in cholesterol elimination and reverse cholesterol transport**							

CYP7A1	A-204C rs3808607	ATV	Indian	A: 59 C: 41	177 DL	Greater LDL reduction (AA)	[14]
		ATV	Chinese Han	GG: 34.6 GT: 50.5 TT: 15.0	107 DL	NS	[22]
		ATV	Chinese Han	AA: 30.4 AC: 47.0 CC: 22.6	181 DL	Greater TG reduction (AA)	[23]
	rs4738687	V	Chinese	TT: 42.4 TC: 42.1 CC: 15.5	386 DL	NS	[24]
	rs2162459	V	Chinese	GG: 31.8 GA: 49.9 AA: 18.3	386 DL	NS	[24]
		ATV	Chinese Han	GG: 23.4 GA: 54.2 AA: 22.4	107 DL	NS	[22]
	rs8192870	ATV	Chinese Han	TT: 51.4 TG: 44.9 GG: 3.7	107 DL	Greater LDL reduction (TT)	[22]
	rs1457042, rs6997473, rs11786580, rs8192879	ATV	Chinese Han	-	107 DL	NS	[22]
	rs3824260	SIM	Chinese Han	GG: 37.5 GA: 44.3 AA: 18.2	420 DL	Lower LDL reduction (AA) Higher HDL reduction (GG)	[53]
CETP	TaqIB	SIM	Thai	B1B1:4 B1B2: 83.6 B2B2: 12.4	225 DL	Greater TC and LDL reduction (B1 carriers)	[47]
LPL	C1421G	RSV	Chinese	CC: 76.7 CG: 22.2 GG: 1.1	386 HCVD, FH, RA	Greater LDL reduction (CG/GG)	[20]
LCAT	rs255052	RSV	Chinese	GG: 82.9 GA: 16.6 AA: 0.5	386 HCVD, FH, RA	Greater LDL reduction (GG)	[20]

**Other genes**							

MMP9	C1562T rs3918242	SIM	Chinese	CC: 71.3 CT: 26.0 TT: 2.7	264 CAD	Greater LDL reduction (TT)	[71]
EL	2037T/C rs3744843	RSV	Chinese	TT: 56.2 CT: 38.8 CC: 5.0	121 CAD	NS	[76]
	2237 G/A rs3744841	RSV	Chinese	GG: 38.0 GA: 49.6 AA: 12.4	121 CAD	NS	[76]
FMO3	Val257Met	RSV	Chinese	GG: 59.5 GA: 34.5 AA: 6.0	386 HCVD, FH, RA	Greater LDL reduction (GG)	[20]
PON1	Q192R	SIM	Chinese	QQ: 23.7 QR: 53.4 RR: 22.9	236 CAD	Greater HDL increment (RR)	[64]
TIMD4-HAVCR1	rs1501908	ATV	Chinese Han	CC: 51.0 CG: 40.1 GG: 8.9	724 H, CAD, IS	Lower TC and LDL level (G allele)	[69]
	rs12522248	ATV	Chinese Han	TT: 70.4 TC: 26.2 CC: 3.4	724 H, CAD, IS	Lower TC and LDL level (C allele)	[69]
	rs2036402	ATV	Chinese Han	TT: 79.3 TC: 19.5 CC: 1.2	724 H, CAD, IS	Lower TC and LDL level (TC)	[69]
LEP	G2548A	SIM	Chinese	AA: 40.1 GA: 54.2 GG: 5.7	212 DL	NS	[61]
LEPR	Q223R	SIM	Chinese	RR: 78.3 QR: 21.7 QQ: 0%	212 DL	Greater TC and TC reduction (RR)	[61]
	A223G	SIM	Chinese	GG: 74.0 AG: 23.4 AA: 2.6	312 CAD	Lower HDL increment (AA)	[62]
FXR	G-1T rs56163822	RSV	Chinese	GG: 81.6 GT: 17.7 TT: 0.7	385 DL	Greater TC and LDL reduction (T allele)	[66]
SCAP	A2386G	RSV	Indian	AA: 36.6 AG: 31.7 GG:31.7	63 DL	Greater TC and LDL reduction (G allele)	[67]
TNF-α	C-857T	U	Japanese	CC: 71.7 CT, TT: 28.3	322 T2DM	Higher LDL and resistant to statin (T allele)	[70]
NCAN/CILP2/PBX4	rs16996148	RSV	Chinese	GG: 81.5 GT: 18.5	386 HCVD, FH, RA	Greater LDL reduction (GG)	[20]

* Drugs studied: ATV, atorvastatin; PRV, pravastatin; RSV, rosuvastatin; SIM, simvastatin; V, various statins; U, unknown statins

# Conference paper

Abbreviations: HDL, high-density lipoprotein; LDL, low-density lipoprotein; TC, total cholesterol TG, triglycerides; NS, not significant; DL, dyslipidemic patients; HCVD, high-risk cardiovascular disease population; FH, familial hypercholesterolemia; RA, rheumatoid arthritis; CAD, coronary artery disease; IS, ischemic stroke; T2DM, Type 2 diabetes mellitus; H, healthy; NA, not available

### Transporters

Transporters are membrane proteins present in cells to regulate the influx of essential nutrients and ions and the efflux of cellular waste and xenobiotics, such as toxins and drugs. The two major superfamilies of transporters that play an essential role in drug disposition are solute carrier (SLC) and ATP-binding cassette (ABC) transporters.

Statins are transported into the liver by the organic anion transporting polypeptide 1B1 (OATP1B1) encoded by *SLCO1B1*, which controls the systemic exposure of many statins and is associated with statin-induced myopathy. Out of the many single nucleotide polymorphisms (SNPs) in *SLCO1B1*, the two most studied are A388G and T521C [9]. Even though the T521C SNP was not associated with the lipid-lowering response of simvastatin and rosuvastatin [10,11,12], Lee et al. [11] reported an association between increased plasma concentrations of rosuvastatin and impaired N-demethylation of rosuvastatin. This was validated in a clinical pharmacokinetic study that reported higher plasma exposure of rosuvastatin and its metabolites among Asian populations in the United States when compared with Caucasians [13]. A study on 177 hypercholesterolemic Indian patients revealed that there was significantly higher LDL-C reduction in patients who were homozygous for the A allele in the A388G SNP when treated with 10 mg of atorvastatin compared to G homozygotes [14]. In contrast, studies among Thai and Chinese hypercholesterolemic patients found no association between A388G SNP and lipid-lowering response [10,11,12]. Interestingly, this SNP has a higher variant allele frequency (57%–75%) compared to wild-type allele frequency (25%–43%) in Chinese, Indian, and Thai populations [10,11,12, 14] than in Caucasians (38.2%) [15]. An intronic SNP, rs4149081 G>A, identified previously in a genome-wide association studies (GWAS) study was studied by Hu et al. [12] among Chinese patients and they reported that rs4149081 G>A SNP was significantly associated with a 4.6% and 4.0% greater reduction of LDL-C compared with those with wild-type alleles in response to rosuvastatin and simvastatin treatment, respectively. However, T89595C and C463A SNPs were not associated with treatment response in Indian and Thai patients treated with simvastatin and atorvastatin, respectively [10, 14].

Multidrug resistance-associated protein 2 (MRP2) also known as ABC sub-family C member 2 (ABCC2) encoded by the *ABCC2* gene, is an efflux transporter expressed on the apical domain of epithelial cells. This transporter has also been associated with hepatobiliary excretion of statins, and SNPs in this gene may have a role in the response to statins [16]. A study on 318 simvastatin-treated Chinese patients revealed that the variant allele of the-24 C>T SNP and female gender were significantly associated with low response of HDL-C elevation upon simvastatin treatment [17]. However, the genotypes were not associated with the differences in TC, TG, LDL-C, or HDL-C among genotypes before and after treatment [17]. On the other hand, in another study, the change in LDL-C was more pronounced among patients who carried both the variant allele for rs717620 in *ABCC2* gene and wild-type allele for rs1128503 in *ABCB1* gene compared to those who carried wild-type genotypes for both of these SNPs [18].

The *ABCB1* gene encodes for P-glycoprotein, which is another efflux pump that transports statins and metabolites from hepatocytes to bile and from renal cells to urine. The two most studied SNPs in *ABCB1* are G2677T (rs2032582) and C3435T (rs1045642). Among Indian atorvastatin-treated patients, carriers of variant allele for G2677T had 3 times more significant reduction of LDL-C as compared to those who carried the wild-type allele (*P* < 0.05) [14]. This study also reported significant association between C3435T polymorphism and LDL-C reduction where carriers of variant allele had greater LDL-C reduction treated with atorvastatin compared to wild-type allele carriers [14].

The *ABCG2* gene that encodes for ABC, sub-family G2 protein or alternatively known as breast cancer resistant protein (BCRP), is mostly found on the plasma membrane and functions as a direct drug efflux pump [19]. A study on 291 Chinese patients reported that C421A*AA homozygotes exhibited 63% and 41% higher mean plasma concentrations of rosuvastatin and its metabolite as compared to heterozygous carriers (CA), while 120% and 99% greater than in those with the homozygous wild-type (CC) genotype when treated with rosuvastatin [11]. Significant correlation was observed between plasma concentrations of rosuvastatin and the percentage reduction in LDL-C (r =−0.194; *P* = 0.001), which disappeared when adjusted for the C421A polymorphism [11]. Hu et al. [20] and Tomlinson et al. [21] reported that this polymorphism is significantly associated with reduction of LDL-C level in a gene-dose dependent manner among Chinese patients, where carriers of homozygous variant genotype (AA) exhibited higher reduction in LDL-C level. On the other hand, for *ABCG5* gene, another study in Indian patients treated with atorvastatin demonstrated no association between *ABCG5* rs6720173 SNP and LDL-C levels [14].

The protein encoded by *ABCG8* functions as a half-transporter to limit intestinal absorption and promote biliary excretion of sterols [19]. An intron 11 SNP rs4148222 was shown to be significantly associated (*P* = 0.046) with baseline levels of HDL-C among Chinese patients treated with atorvastatin [22]. Another study among Chinese patients treated with atorvastatin found no association between *ABCG8* C1199A polymorphism and the lipid-lowering response of atorvastatin, yet the response appeared to be affected by synergistic effect of *CYP7A1* and *ABCG8* polymorphisms [23]. Greater TG and TC reduction (*P* = 0.001 and *P* = 0.009, respectively) were observed in patients with the *CYP7A1* A-204C homozygous wild-type genotype (AA) compared to the homozygous variant genotype (CC) [23]. A Taiwanese study found that patients carrying the ATTATCGAC haplotype demonstrated greater reduction of LDL-C [24]. However, an Indian study did not show any association between the D18H variant and LDL-C levels [25].

The *ABCA1* gene encodes for a human transporter known as cholesterol efflux regulatory protein (CERP). The KK homozygotes of the R219K SNP in *ABCA1* exhibited significantly higher increase in HDL-C level compared to wild-type homozygotes RR in Chinese patients treated with pravastatin [26].

### Cytochrome P450 and related enzymes

Genetic polymorphisms in cytochrome P450 (CYP) genes have been associated with the efficacy of selected statins by influencing their hepatic metabolism. The CYP450 enzymes catalyze the metabolism of most statins, except pravastatin and rosuvastatin (**Figure 2**). Atorvastatin and simvastatin are metabolized by the CYP3A4 isoenzyme and fluvastatin via CYP2C9 [27]. In addition, metabolism of statins may be mediated by other CYP450 isoenzymes, including CYP2D6 [28]. A meta-analysis of population-scale sequencing project showed that the distribution of CYP alleles, including *CYP3A4*, *CYP3A5*, *CYP2C9*, *CYP2C19*, and *CYP2D6*, differs considerably between different ethnic groups (Europeans, Africans, East Asians, South Asians, and admixed Americans) [29].

Hu et al. [30] found no significant association between CYP3A4*1G and CYP3A4*22, along with other gene polymorphisms (CYP3A5*3 and *PPARA* rs4823613 A>G), with response to 40 mg simvastatin in 273 Chinese patients with hypercholesterolemia. However, a prospective study by Gao et al. [31] reported a CYP3A4*1G-dose-dependent reduction of TC in hyperlipidemic patients treated with atorvastatin but not simvastatin. Peng et al. [32] also showed that Chinese patients with ischemic stroke who carried the rs2242480*C allele had better lipid-lowering effect with atorvastatin treatment compared to those who were TT homozygotes. However, this association did not survive Bonferroni correction [32]. Hypercholesterolemic Indian patients with the wild-type genotype of rs2740574 (*CYP3A4*) demonstrated significant reductions in LDL-C in response to atorvastatin therapy [14]. However, results have been inconsistent with the *CYP2C9* polymorphisms. Lin et al. [33] reported that Chinese patients carrying the CYP2C9*1/*3 or CYP2C9*3/*3 genotypes showed a significant lipid-lowering efficacy when treated with rosuvastatin, with higher TC and LDL-C reduction compared to patients with the wild-type genotype. However, other *CYP2C9* SNPs (rs1934967; rs1057910) were not associated with statin treatment response in Chinese patients [11, 24].

Studies found no significant associations between statins response in LDL-C reduction and *CYP2C19* polymorphisms (rs10786172; rs4244285; rs4986893) among Chinese patients [11, 24, 32]. However, Finkelman et al. [34] reported that changes in lipid profiles from baseline for poor metabolizers (PMs: *2*2, *2*3, *3*3) and extensive metabolizers (EMs: *1*1, *1*2, *1*3) of *CYP2C19* were comparable except for TG among healthy Taiwanese receiving multiple doses of 20 mg rosuvastatin. The PMs demonstrated a greater TG reduction compared to EMs.

Li et al. [35] investigated the effects of the lipid-lowering effect of simvastatin and CYP2D6*10 in 200 Chinese Ningxia Hui patients with hyperlipidemia. The lipid-lowering effects of simvastatin 20 mg daily, after 8 weeks of treatment, was increased significantly with the genotype CC, leading to the authors recommending that patients carrying the CC genotype in this population should be prescribed lower doses of simvastatin compared to those with CT and TT genotypes. Additionally, Chinese patients carrying the rs1065852*G allele showed better lipid-lowering effect with atorvastatin therapy [32].

CYP3A5 is another CYP3A family enzyme that is involved in metabolism of simvastatin and atorvastatin. However, no studies found any significant associations between *CYP3A5* polymorphisms (rs776746, rs4646450, rs3800959, and rs776746) with the lipid-lowering effects of simvastatin or atorvastatin among Chinese or Indian patients [14, 22, 30, 36].

*CYP3AP1* is a pseudogene in the human CYP3A gene, which can play a vital role in gene conversion and recombination with a functional gene. Li et al. [37] investigated the lipid-lowering response of atorvastatin and simvastatin with CYP3AP1*3 (−44G>A) polymorphisms in 379 Chinese Han hyperlipidemic patients. There was a greater LDL-C reduction in response to simvastatin therapy in women who were CYP3API*3/*3 carriers compared to CYP3API*1/*3 carriers. In the atorvastatin group, the TC level was significantly reduced in women who were CYP3API*3/*3 carriers than in CYP3API*1/*3 carriers [37].

P450 oxidoreductase (POR) plays an essential role for CYP450 activity by transferring electrons from nicotinamide adenine dinucleotide phosphate (NADPH) to CYP3A enzymes, which metabolize various statins, including atorvastatin [38]. Wei and Zhang [36] investigated the association of POR*28 polymorphisms and the lipid-lowering effects of atorvastatin (20 mg/d for 4 weeks) in 179 Chinese patients with hyperlipidemia. Homozygotes for the T allele showed significantly lower LDL-C levels than homozygotes for the C allele (wild-type) after atorvastatin treatment. After adjustment for age, sex, and body mass index, CYP3A5 non-expressors who were POR*28 wild-type homozygotes (CC) presented with significantly higher mean TC and LDL-C levels than those who were POR*28 variant homozygotes (TT) and heterozygotes (CT) at baseline and after atorvastatin treatment [36]. This indicates that POR*28 SNPs are associated with significant increases in plasma lipids in non-expressors of *CYP3A5*.

### Apolipoprotein genes

Apolipoproteins have a role in regulating lipoprotein metabolism [39]. Polymorphisms in several apolipoproteins, such as *APOE*, *APOA5*, and *APOB*, have been reported to affect statin response. Apolipoprotein A-V (APOA5) is particularly involved in the metabolism of TG-rich lipoproteins [40], and has previously been shown to be associated with a high degree of interindividual variation in serum TG [41,42,43]. A study by Hua et al. [44] in 195 Chinese patients with treatment-naïve dyslipidemia explored the association of *APO5* rs662799 (T-1131C) SNP following a 12-week randomized treatment of three statins: rosuvastatin, atorvastatin, or simvastatin. Patients carrying the rs662799*TT genotype exhibited greater LDL-C reduction regardless of whichever statin they were being treated with. However, this genotype was only correlated with HDL-C and TG in atorvastatin- and simvastatin-treated groups [44]. Interestingly, this lack of association of rosuvastatin with this SNP was validated by Hu et al. [45] in their cohort of Chinese patients with hyperlipidemia.

Apolipoprotein E (APOE) is expressed in the brain and the liver and is a ligand for the LDL receptor (LDLR) [46]. Similar to *APOA5* polymorphism, Hu et al. [45] reported that there was no significant association between *APOE* E2/E3/E4 polymorphisms and response to rosuvastatin among Chinese patients with hyperlipidemia. Thai hypercholesterolemic patients who were *APOE2* and *APOE3* carriers had significantly greater TC and LDL-C reduction compared to *APOE4* carriers after 3 months of simvastatin treatment [47]. On the other hand, a study by Hu et al. [20] found that the LDL-C reduction after at least 4 weeks of rosuvastatin 10 mg daily treatment was significantly higher among carriers of the rs4420638*A allele within the APOE/C-I/C-IV/C-II gene cluster.

Liu et al. [48] found that there was a significant reduction in TC, LDL-C, and APOB levels after 12 weeks of pravastatin therapy only among Chinese hyperlipidemia patients expressing the *APOA1* (G75A) AA and GA genotypes.

### *HMGCR* gene

3-Hydroxy-3-methyl-glutaryl-coenzyme A reductase (HMGCR) is the rate-controlling enzyme for cholesterol synthesis via the mevalonate pathway, which is the target enzyme of statins [49].

Chien et al. [24] explored the association of two *HMGCR* SNPs (rs12916 and rs3931914) and efficacy after 9 months of treatment with various statins, including atorvastatin, simvastatin, and pravastatin, in 386 Chinese patients with hyperlipidemia. Patients who carried the rs12916*CT and TT genotypes showed a significant greater reduction in LDL-C than the CC homozygotes after correction for multiple tests in an additive model [24]. Additionally, the CCGTCCA haplotype was associated with a significant increase in LDL-C. However, there was no significant association with rs3931914. Among 24 healthy Koreans, Chung et al. [50] noticed that the *HMGCR* rs3846662 GG genotype demonstrated significantly higher levels of LDL-C at baseline and all observation times (7–28 d after atorvastatin therapy), with mean differences of 18%–33%.

### LDLR and related genes

Hu et al. [20] reported that *LDLR* rs1433099 polymorphism was one of the predictors for the LDL-C changes in response to rosuvastatin in 386 Chinese patients. Individuals with the TT genotype demonstrated a significantly lower reduction in LDL-C level compared to those with the CC and CT genotypes after adjustment for age, sex, and having familial hypercholesterolemia, but not significant after Bonferroni correction [20].

In addition to LDL receptors, proprotein convertase subtilisin/kexin type 9 (*PCSK9*) regulates levels of plasma LDL by binding to the LDLRs and directing it to endosomes or lysosomes for destruction [51]. Reduction in LDLRs decreases clearance of LDL-C and ultimately leads to accumulation of LDL-C in the blood circulation. Wanmasae et al. [47] investigated the variation in three *PCSK9* SNPs (R46L, I474V, and E670G) on the lipid-lowering effects of simvastatin in 223 Thai hypercholesterolemic patients. There was a significant reduction of LDL-C in *PCSK9* 474IV carriers compared to 474II carriers, but no significant difference for TC, TG, and HDL-C. However, the association between *PCSK9* R46L and E670G and simvastatin response was not analyzed due to low frequencies of minor alleles [47].

Sterol regulatory element-binding transcription factor 1 (SREBF1) is the transcription factor that regulates the expression of hepatic LDLR [24]. A study explored the association of *SREBF1* rs9902941 polymorphisms and response to various statins among 386 Chinese patients with hyperlipidemia. Those subjects carrying minor T alleles had significantly higher reduction of LDL-C levels compared to those who were CC homozygous [24].

### Enzymes involved in cholesterol elimination and reverse cholesterol transport

Cholesterol 7α-hydroxylase (CYP7A1) is the rate-limiting enzyme in bile acid production, which is essential for cholesterol elimination from the body. Hence, genetic variants in the gene may indirectly affect an individual's sensitivity to the lipid-lowering effects of statins [52]. Among the Chinese Han hypercholesterolemia patients whose LDL levels were recorded in pursuance of atorvastatin treatment, a significantly greater reduction was, among all the 7 variants studied, demonstrated only for rs8192870*T homozygotes [22]. A similar effect was also seen with Indian and Chinese patients treated with atorvastatin who were homozygous for the rs3808607*A allele [14, 23]. However, the study by Chien et al. [24] did not find any associations with the two SNPs, rs4738687 and rs2162459, in their cohort of Chinese patients. Recently, Liu et al. [53] reported that *CYP7A1* rs3824260 is associated to different responses to simvastatin among those Chinese hypercholesterolemia patients. They revealed that AA homozygotes tend to have low response to LDL-C reduction, whereas GG homozygotes were at a higher risk of HDL-C reduction after 12 weeks of simvastatin therapy [53].

Cholesterol ester transfer protein (CETP) encoded by the *CETP* gene is a plasma protein that facilitates the transfer of lipids between HDL and TG-rich lipoproteins. The most studied SNP in *CETP* is the *TaqIB* variant, which has been associated with statin response [16]. Previous studies reported that the B1 allele is associated with increased CETP concentrations as well as activity and thus low HDL-C levels while the opposite was reported for the B2 allele [54,55,56]. In 225 hypercholesterolemic Thai patients treated with simvastatin, better lipid-lowering effect with higher reductions in TC and LDL-C was observed among B1 carriers (B1B1 + B1B2) compared to the B2 homozygotes [47].

Lipoprotein Lipase (LPL) plays a role in lipid metabolism by catalyzing the hydrolysis of triacylglycerol in chylomicrons and very low-density lipoproteins (VLDL) for tissue utilization [57]. Hu et al. [20] reported that Chinese patients with the C1421G CC genotype demonstrated a significantly lower reduction in LDL-C levels compared to those with the CG or GG genotypes in response to rosuvastatin treatment.

Lecithin-cholesterol acyltransferase (LCAT) plays a key role in cholesterol transport and removal by catalyzing the conversion of cholesterol and lecithin to cholesteryl esters and lysophosphatidylcholines on the surface of HDLs, which is a crucial step in the maturation of HDLs [58]. A study investigated the association of *LCAT* rs255052 with the lipid-lowering effect of rosuvastatin in 386 Chinese patients and found that the GG genotype carriers demonstrated a greater reduction in LDL-C levels than those carrying the A allele [20].

### Other genes involved in statins response

In addition to the aforementioned gene variations affecting pharmacokinetics and pharmacodynamics of statins, other gene polymorphisms were also studied among Asian populations. Flavin-containing monooxygenase 3 (FMO3) catalyzes the oxygenation of various compounds and plays a regulatory role in cholesterol metabolism [59]. A study revealed that the *FMO3* V257M variation was one of the predictors of LDL-C response in Chinese patients on rosuvastatin therapy. Patients with the V257M GG genotype showed higher reductions in LDL-C levels in comparison to those with the GA and AA genotypes [20].

Leptin may also be involved in regulating cholesterol metabolism via other mechanisms, such as downregulating activity of HMG-CoA reductase and upregulating activity of sterol and cholesterol hydroxylases to decease VLDL levels [60]. Li et al. [61] investigated the association between *LEP* G2548A and *LEPR* Q223R polymorphisms and efficacy of simvastatin in 212 Chinese patients with primary hyperlipidemia. Patients carrying the Q223R RR genotypes had significantly higher reductions in TG and TC levels than those with the QR genotypes among poor respondents. However, this significance did not survive Bonferroni correction [61]. Sun et al. [62] observed that the increment of HDL-C levels was significantly lesser among patients who were AA homozygotes than those with the GG genotype. However, there was no significant association between *LEP* G2548A SNP with treatment response [61].

Paraoxonase 1 (PON1) is an HDL-associated enzyme involved in reducing lipid peroxide accumulation on LDL, suggestive of a protective role against cardiovascular disease [63]. Fu et al. [64] noticed that there was significant association between plasma HDL-C changes with *PON1* Q192R polymorphism among 236 Chinese patients with coronary heart disease receiving simvastatin therapy. Patients with the RR genotype presented with greater increment in HDL-C levels in response to simvastatin therapy, than patients with the QR or QQ genotypes [64].

Farnesoid X receptor (FXR), the bile acid-activated nuclear receptor with an essential role in lipid and carbohydrate metabolism, is a regulator of drug transporters involved in statin disposition, including SLCO1B1 [65]. A study investigated the impact of *FXR* G-1T polymorphism on the lipid-lowering response of rosuvastatin among Chinese patients with hyperlipidemia. The authors observed that individuals who carried the T allele (GT or TT) had greater reductions in LDL-C and TC compared to those who were homozygous for the G allele [66]. Sterol regulatory element-binding factors cleavage activating protein (SCAP) has a regulatory role in lipid homeostasis. A study showed that Indian patients carrying the *SCAP* A2386G*G allele presented with significant reductions in their TC and LDL-C levels in comparison to those homozygous for the *A allele [67].

T-cell immunoglobulin and mucin domain 4 gene (*TIMD4*) and hepatitis A virus cellular receptor 1 gene (*HAVCR1*; also known as *TIMD1*) are genes essential in regulating immune responses. *HAVCR1* is expressed on T-helper cells and co-stimulates T-cell activation, whereas *TIMD4* is expressed on antigen-presenting cells and mediates phagocytosis of apoptotic cells [68]. Zhang et al. [69] investigated the effect of three *TIMD4-HAVCR1* SNPs (rs12522248, rs1501908, and rs2036402) on the lipid-lowering effect of atorvastatin in a population of 724 individuals of southern Chinese Han ethnicity. They demonstrated that patients who carried the rs1501908*G and rs12522248*C alleles had lower TC and LDL-C levels than those carrying the rs1501908*C and rs12522248*T alleles, respectively. Additionally, patients with the rs2036402*TC genotype presented with lower TC and LDL-C levels than those with the TT genotype. Interestingly, patients with the rs1501908*CC, rs12522248*TT, or rs2036402*TT genotypes presented with lower APOA1 levels than other respective genotypes after atorvastatin treatment [69]. These associations highlighted that atorvastatin had significantly higher efficacy in reducing TC and LDL-C levels in patients who carried the *TIMD4-HAVCR1* rs1501908*G and rs12522248*C alleles. On the other hand, Takahashi et al. [70] reported that the C-857T polymorphism in the promoter region of the *TNF-*α gene was associated with significantly higher LDL-C levels and lower cholesterol-lowering effects in patients who were carriers of the T allele in comparison to non-T carrier when treated with statins.

Matrix metalloproteinase 9 (MMP9) has been shown to be associated in the pathogenesis and progression of cardiovascular diseases [71]. Xu et al. [71] investigated the association of *MMP9* C1562T polymorphism on the lipid-lowering effect of simvastatin in 264 Chinese patients with coronary artery disease. Their results showed that the reduction of LDL-C with simvastatin therapy was significantly greater among patients who were TT homozygotes compared to CC homozygotes [71]. Polymorphism on neurocan/cartilage intermediate layer protein 2/pre-B-cell leukemia homeobox 4 (*NCAN/CILP2/PBX4*) gene located on chromosome 19 has been shown to be associated with different serum lipid phenotypes among Europeans and Asians [72,73,74]. Hu et al. [20] investigated the association of rs16996148 with the lipid-lowering effect of rosuvastatin in Chinese patients and observed that patients who were homozygous for the rs16996148*G allele had greater LDL-C reduction than the GT heterozygotes.

Endothelial lipase (EL) has a role in modulating lipid metabolism by lowering HDL-C levels [75]. However, Cai et al. [76] reported that there was no significant effect of *EL* 2037 T/C and 2237 G/A polymorphisms on the lipid-lowering effects of rosuvastatin (10 mg/d for 4–8 weeks) in 121 Chinese with coronary artery disease.

### Susceptibility to statin-induced ADRs

Five papers investigated associations between various genetic variants with statin-induced ADRs, studying the effects of either just one statin [33, 77] or multiple statins [78,79,80] (**Table 2**). As the studies used different methods in determining statin-induced ADRs and studied different genes, direct comparisons to derive a definitive conclusion were not possible. The most robust and well-defined method of defining statin-induced ADR was adopted in the research of Sai et al. [78]. In this study, statin-related myopathy was presented as three distinct categories: myalgia (defined by creatine kinase [CK] value <3-fold of the upper limit of normal with muscle symptoms), myositis (defined by CK value between 3- and 10-fold of the upper limit of normal (ULN) with muscle symptoms), and rhabdomyolysis (defined by CK value >10-fold of the ULN with muscle symptoms) [78]. The remaining four studies depended on either patient self-reported ADR symptoms or checking for ADRs in medical records.

**Table 2.** Genetic polymorphisms related to susceptibility to statin-induced adverse drug reactions among Asians

**Gene**	**Variant**	Statin^*^	**Ethnicity**	**Allele/Genotype/Haplotype frequency (%)**	**Study population**	**Adverse drug reaction**	**Reference**
SLCO1B1	T521C (rs4149056)	V	Chinese	TT: 68.2 TC, CC: 31.8	148 CAD	Higher risk for myopathy (TC/CC treated with RSV); NS for ATV and SIM	[79]
		V	Chinese (Hakka)	NA	47 H	NS	[80]
		V	Japanese	TT: 78.8 TC, CC: 21.2	52 SRM	NS	[78]
	A388G (rs2306283)	V	Chinese	AA: 6.8 GA: 31.1 GG: 62.2	148 CAD	NS	[79]
APOE	C526T (rs7412)	V	Chinese	CC: 87.2 TC, TT: 12.8	148 CAD	NS	[79]
	T388C (rs429358)	V	Chinese	TT: 16.2 TC, CC: 83.8	148 CAD	NS	[79]
CYP2D6	^*^1	RSV	Chinese	NA	16 DL	NS	[33]
CYP3A5	A6986G (rs776746)	V	Chinese	AA: 5.4 GA: 42.6	148 CAD	NS	[79]
LEP	G2548A	SIM	Chinese	AA: 53.0 GA:40.9 GG: 6.1	587 DL	CK elevation (AA)	[77]
LEPR	Q223R	SIM	Chinese	RR: 77.2 RQ:21.6 QQ: 1.2	587 DL	CK elevation (RQ/QQ)	[77]
HLA-DRB1	^*^04:06	V	Japanese	Carrier: 17.3	52 SRM	Higher risk for myopathy	[78]
RYR2	rs2819742	V	Japanese	TT: 98.1 TC, CC: 1.9	52 SRM	NS	[78]
GATM	rs9806699	V	Japanese	AA: 78.6 GA, GG: 21.4	52 SRM	NS	[78]

* Drugs studied: ATV, atorvastatin; RSV, rosuvastatin; SIM, simvastatin; V, various statins

NS, not significant; DL, dyslipidemic patients; CAD, coronary artery disease; H, healthy; NA, not available; CK, creatine kinase; SRM, statin-related myopathy

Sai et al. [78] compared genetic variants in three candidate genes (*SLCO1B1*, *RYR2*, and *GATM*) and four *HLA* genes in 52 patients who had experienced statin-induced myopathies and 2878 healthy controls. Of the variants tested, only HLA-DRB1*04:06 was statistically significant after Bonferroni correction. However, HLA-DRB1*11:01 was previously identified to be significantly associated with antibody positive myopathy in Caucasians and African patients [81]. These differences could have been due to the differences in allele frequencies between the Japanese and Caucasian/African populations.

The study with the largest cohort classified statin-induced myopathy based on CK levels and geographic location in China (392 patients from Dongzhi and 195 patients from Beijing) over two time points, specifically after 4 weeks’ and 8 weeks’ treatment with simvastatin [77]. Jiang et al. [77] only found significant elevation of CK levels among patients in Dongzhi with either the AA genotype in the *LEP* G2548A variant and RR or QQ genotypes of the *LEPR* Q233R variant. However, this finding was not replicated in patients from Beijing.

On the other hand, Liu et al. [79] reported that the risk for myotoxicity was significantly higher among patients who were C allele carriers of *SLCO1B1* T521C taking rosuvastatin. However, this effect was statin-dependent and was not observed in patients taking either atorvastatin or simvastatin [79]. Controversially, Zhong et al. [80] reported that healthy Chinese Hakka subjects with the CC genotype (n = 47) did not experience any statin-induced myopathy.

## Discussion

Although statins are generally effective and well-tolerated, there are interindividual differences that contribute to decreased efficacy and an increase in susceptibility to adverse effects. Ethnicity of patients is one of the factors that affect the efficacy and toxicity of statin therapy, and it has become an integral component of pharmacogenomics studies [82]. A meta-analysis of population-scale sequencing projects revealed that there are inter-ethnic differences in CYP450 polymorphisms between populations, which are crucial for personalized drug therapy and healthcare programs [29].

Asia is the largest and most diverse continent with several heterogenous populations with significant implications for pharmacogenomics. Malaysia and Singapore are countries with multiple ethnicities, predominantly Malay, Chinese, and Indian. These countries provide noticeable examples of pharmacogenomic diversity across different ethnicities within the population. Ho et al. [83] reported that there were significant inter-ethnic differences in allele frequencies across *SLCO1B1* SNPs among healthy Malay, Chinese, and Indian Singaporeans. Specifically, they showed that the allele frequencies in Indian Singaporeans were significantly different to those found in Chinese (*P* < 0.001) and Malay Singaporeans (*P* < 0.001). The genetic differences among ethnicities have received greater attention from researchers, prompting them to further investigate interindividual differences in the drug response, which is important in personalized drug therapy.

Several candidate genes have been studied to investigate their effects on efficacy and safety of statins among Asian populations, including transmembrane transporters, CYP450 isoenzymes, and apolipoproteins. *SLCO1B1* was most extensively investigated among Asian populations due to its strong association with statin-induced myopathy among Caucasians as reported in the STRENGTH and SEARCH trials [84, 85]. Consecutively, *SLCO1B1* T521C (rs4149056) SNP testing and genotype-guided dosing have been implemented for simvastatin in some countries [86]. However, the presence of controversial findings obscures the implementation of this guideline among Asian populations. The rs4149056 SNP has not been found to be significantly associated with susceptibility to statin-induced ADRs in Japanese, Chinese, or South Indian patients [78, 80, 87]. However, it has been reported to be significant among Chinese patients [79], alluding to pharmacogenomic variability being evident across different ethnicities. On the other hand, a recent Malaysian study reported that *SLCO1B1* T89595C (rs4363657) polymorphism was a significant risk factor for statin-induced myopathy in heterozygote carriers compared to wild-type and variant homozygotes. However, this polymorphism was not found to be significantly different between Malay, Chinese, and Indian ethnicities [88]. The applicability of these findings is limited by the small sample size involved in this pilot study.

Several clinical trials investigating the statin treatment response in Western population had underrepresentation of patients of Asian ethnicities. In this review, most of the studies have been conducted among Chinese patients and to a lesser extent among Indian, Thai, and Japanese patients. There are limited large-scale pharmacogenetic studies conducted among patients of non-Caucasian ethnicities to explore the effect of ethnicity on the efficacy and susceptibility of adverse effects to statin treatment. The US Food and Drug Administration (FDA) [89] recommends dosage reduction of rosuvastatin in Asian patients stating in their warning that Asian patients are at higher risk for rhabdomyolysis. The FDA has advised that rosuvastatin dose should be halved in Asian patients because of the 2-fold increase in rosuvastatin exposure when compared to Caucasian patients [89]. Nevertheless, a recent study that evaluated the effect of *SLCO1B1* and *ABCG2* gene polymorphisms on rosuvastatin pharmacokinetics in healthy Asian and Caucasian subjects in the USA reported that there was no difference in rosuvastatin exposure between Asian and Caucasian subjects when all subjects were wild-type carriers for SLCO1B1*1a and *ABCG2* C421A [90]. However, this finding is less conclusive due to the study's small sample size with only 15 subjects of East Asian descent. Therefore, the effect of ethnic differences in rosuvastatin treatment response remains unclear.

This review has identified a gap in our current knowledge in understanding the underlying genetic basis of variability in statin response and predisposition to adverse effects in the many ethnic groups in Asia. Conflicting findings from various studies among Chinese patients have also been reported. This discrepancy could be attributed to other non-genetic confounding factors, such as age, gender, body mass index, diet, concomitant medications, lifestyle and environmental factors that could also influence efficacy, and risk of ADRs of statins [91]. These factors should be considered in study design. In addition, most studies have investigated a small number of genetic variants. However, this scoping review did not analyze the quality of included studies. This scoping review only focused on original research articles published in English articles, and therefore it is possible that some relevant studies published in other languages could have been missed and not included in this study.

## Conclusion

Pharmacogenetic or pharmacogenomic studies provide a fundamental approach toward personalized medicine in statin treatment outcomes by exploring the individual variability in response to statin treatment. Although statins are generally effective and well-tolerated, there are interindividual differences that contribute to a decreased efficacy and an increased risk of adverse effects to statin therapy. However, various confounding factors should be considered in study design to explore the true effect of ethnicity on statin treatment outcomes. Due to paucity of studies on patients of various Asian ethnicities and discrepancies in the findings from various published papers, continued research pertaining to ethnicity, the candidate genes, and their association with statin efficacy and risk of ADRs is needed to underpin the potential value of personalized medicine in statin therapy.
